# Central Nervous System Mold Infections in Children with Hematological Malignancies: Advances in Diagnosis and Treatment

**DOI:** 10.3390/jof7030168

**Published:** 2021-02-26

**Authors:** Marie Luckowitsch, Henriette Rudolph, Konrad Bochennek, Luciana Porto, Thomas Lehrnbecher

**Affiliations:** 1 Division of Pediatric Hematology and Oncology, Hospital for Children and Adolescents, University Hospital Frankfurt, Goethe University, 60590 Frankfurt am Main, Germany; Marie.Luckowitsch@kgu.de (M.L.); Henriette.Rudolph@kgu.de (H.R.); Konrad.Bochennek@kgu.de (K.B.); 2 Institute for Neuroradiology, University Hospital Frankfurt, Goethe University, 60590 Frankfurt am Main, Germany; Luciana.Porto@kgu.de

**Keywords:** central nervous system, invasive fungal disease, child, mold, *Aspergillus*, mucormycetes

## Abstract

The incidence of invasive mold disease (IMD) has significantly increased over the last decades, and IMD of the central nervous system (CNS) is a particularly severe form of this infection. Solid data on the incidence of CNS IMD in the pediatric setting are lacking, in which *Aspergillus* spp. is the most prevalent pathogen, followed by mucorales. CNS IMD is difficult to diagnose, and although imaging tools such as magnetic resonance imaging have considerably improved, these techniques are still unspecific. As microscopy and culture have a low sensitivity, non-culture-based assays such as the detection of fungal antigens (e.g., galactomannan or beta-D-glucan) or the detection of fungal nucleic acids by molecular assays need to be validated in children with suspected CNS IMD. New and potent antifungal compounds helped to improve outcome of CNS IMD, but not all agents are approved for children and a pediatric dosage has not been established. Therefore, studies have to rapidly evaluate dosage, safety and efficacy of antifungal compounds in the pediatric setting. This review will summarize the current knowledge on diagnostic tools and on the management of CNS IMD with a focus on pediatric patients.

## 1. Introduction

The incidence of invasive fungal disease (IFD) has significantly increased over the last decades, as it was shown in two sequential autopsy studies performed in a large University hospital in Germany covering the time period of 1977–2005 [1,2]. Corroborating an analysis of 11 autopsy reports, these autopsy studies identified *Aspergillus* spp. as the most common pathogen, followed by *Candida* spp. and mucorales [3]. The clinical impact of invasive aspergillosis was demonstrated by a retrospective cohort study in the US analyzing 666 cases of invasive aspergillosis among 152,231 immunocompromised children [4]. Invasive aspergillosis not only significantly increased the length of hospital stay and costs, but was also associated with higher mortality independently from the underlying malignancy.

Invasive mold infections mainly affect the lung, but may also occur in other sites such as liver, kidney, bones, or the central nervous system (CNS). The CNS involvement is a particularly severe form of fungal infection, which is not only difficult to diagnose but also difficult to treat. Mortality of invasive mold disease of the CNS (CNS IMD) is unacceptably high, and those patients who survive often suffer from significant and permanent neurological sequelae. Here, we review the current knowledge on diagnostic tools and the management of CNS IMD with a focus on pediatric patients.

## 2. Epidemiology and Risk Groups

Both epidemiology of and risk factors for IFD in general have been analyzed in a number of studies. In a retrospective pediatric analysis, highest incidence rates for IFD were found for patients undergoing allogeneic hematopoietic stem cell transplantation (HSCT) (13.9%), de novo (10%) and relapsed (17.9%) acute myeloid leukemia (AML), which corroborate the data of a 2-year prospective analysis in three major pediatric centers [5,6]. A systematic review including 22 studies found that the underlying disease (e.g., AML, high risk acute lymphoblastic leukemia (ALL), relapsed acute leukemia), allogeneic HSCT (in particular with acute and chronic graft-versus-host-disease), prolonged neutropenia (>10 days), high-dose steroids, and increasing age were the most relevant risk factors for IFD in children [7]. Outside the clinical setting of cancer patients, diabetes, low birth weight, malnutrition, and liver diseases are well described risk factors for mucormycosis [8].

Whereas incidence rates of IFD are quite consistent across most of the studies, the incidence of CNS IFD is less clear [9]. In a prospective surveillance study in adults (median age, 40 years), 232 IFDs were identified, 113 in patients with hematological malignancies and 119 in patients without hematological malignancies [10]. Among the patients with hematological malignancies and IFD, invasive aspergillosis occurred in 86 patients (76.1%; 18 patients suffered from *Aspergillus fumigatus*, 14 from *A. flavus*, two from *A. terreus*, and one each from *A. niger*, *A. flavipes, A. nidulans*, or from more than one species; in 48 patients, the species of *Aspergillus* could not be identified). In 11patients (9.7%), *Fusarium* spp. was isolated (*Fusarium solani* in three patients, *Fusarium proliferatum* and *Fusarium verticilloides* in one patient each), and mucormycosis was diagnosed in seven patients (6.2%; *Rhizopus oryzae* in three patients, and *Absidia corymbifera*, *Rhizomucor pusillus*, *Mucor* spp., and *Rhizopus* spp. in one patient each). In the 113 patients with hematological malignancies, 16 patients with CNS IMD (14.1%) were observed, whereas this was the case in 13 out of the 119 patients without hematological malignancies (10.9%). Unfortunately, the authors did not report on the pathogens identified in the patients with CNS IMD. In a multicenter retrospective study analyzing pediatric patients (median age, 9.3 years), 127 IFDs were diagnosed in 123 out of 2183 patients (5.6%), and the CNS was involved in only four of the 123 patients (3.3%) [5]. The pathogens in these children have not been reported. In an analysis of 25 cases of mucormycosis occurring in 1136 prospectively registered children with acute leukemia (2.2%), 23 patients (59%) had a rhinocerebral pattern of infection, eight of them with adjacent spread to the CNS [11]. *Rhizopus* spp. was the most common causative pathogen (46% of patients). Whereas in a large case series of 89 proven or probable CNS IFD in mostly adult patients (median age, 40 years) *Aspergillus* spp. was the most common pathogen (69%), followed by mucormycetes (22%), *Cryptococcus* spp. (4%) and *Fusarium* spp. (2%), data in the pediatric setting are lacking [12].

## 3. Pathogenesis

**Figure 1:**
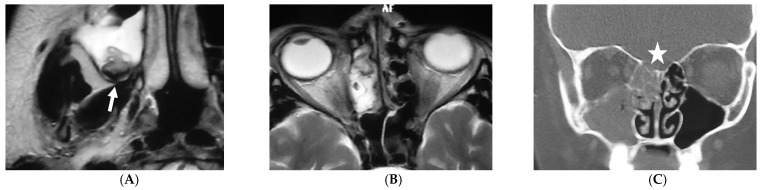
A 7-year-old girl with relapse of acute lymphoblastic leukemia (ALL) and biopsy-proven aspergillosis of paranasal sinuses. The patient has deceased. Axial T2-weighted magnetic resonance images (MRI) (**A** and **B**) show abnormal soft tissue filling the ethmoidal cells and in maxillary sinus on the right with T2-hypointensity (straight arrows). Coronal computerized tomography (CT) (**C**) shows destruction of the sinus walls and the cribriform plate (star) with extension into the anterior cranial fossa. Follow-up images (not shown) showed multiple parenchymal bleedings on the frontal lobes.

Molds are ubiquitous organisms which can be found in soil, water, and decaying vegetation [13]. The portal of entry of these pathogens is usually the respiratory tract, with subsequent hematogenous dissemination to the CNS. Direct inoculation of CNS or paraspinal tissue as a result of surgery or trauma may occur, or fungi may affect sinuses or mastoids and directly expand to the CNS (Figure 1) [14,15]. Whereas yeast are able to enter the CNS directly through the blood–brain barrier either trans-cellularly, para-cellularly or inside infected phagocytes (“Trojan horse mechanism”), the exact mechanism of the blood–brain barrier penetration of molds is not well understood to date [14,15,16,17]. It was speculated that mycotoxins such as gliotoxin produced by *A. fumigatus* exhibit various effects which ultimately lead to intracerebral invasion of the fungus. For example, mycotoxins are immunosuppressive and reduce the opsonization of the conidia and inhibit phagocytosis. Mycotoxins also damage microglia, which exhibits phagocytic abilities and is the major surveillance of the CNS against the fungus [14,16,18,19]. Similarly, fumonisin B1, a mycotoxin produced by *Fusarium* spp., damages the microglia and leads to neuronal axon demyelination [20]. However, a better understanding of the exact pathomechanisms is urgently needed which might also help to develop new approaches in preventing and treating CNS IMD.

## 4. Clinical Presentation

The clinical symptoms of CNS IMD depend on the site of involvement and are often unspecific (e.g., fever refractory to antibiotic treatment), so that early diagnosis is difficult. First signs of rhino-cerebral IMD are commonly headache, periorbital redness and tenderness, and protrusion of the eye [21]. Depending on the extent and the localization, intracerebral lesions may result in cranial nerve palsy, seizures, hallucination, and somnolence.

An analysis in 89 adult patients (median age, 40 years) reported that the most common presenting symptom of CNS IFD was fever (65%) followed by headache (26%) [12]. Focal neurological symptoms (e.g., aphasia, diplopia) were seen in 48%, and seizures occurred in 18%. Interestingly, in a study on CNS IMD in 29 children, 21 (72%) presented with CNS related symptoms, whereas eight (28%) were neurologically asymptomatic and CNS IFD was detected by diagnostic work-up for pulmonary or liver fungal infection or during routine CNS imaging for assessing tumor response [22]. Common neurological symptoms were somnolence and cerebral palsy (each 24%), followed by headache (21%). In one patient each, impaired vision, aphasia, ataxia, hallucination, paresthesia, and seizures were reported. These data indicate that in patients at risk for IFD, further diagnostic work-up including imaging studies with magnetic resonance imaging (MRI) of the brain should be considered in patients with any CNS related symptom or in those with proven or probable IMD outside the CNS [23].

## 5. Diagnostic Imaging

Imaging, in particular MRI, is an important diagnostic tool for identification and monitoring of CNS IMD. MRI is the preferable modality in the pediatric setting due to its sensitivity and lack of radiation, but it may need general anesthesia, especially in young children. Unfortunately, the interpretation of MRI findings in immunocompromised patients with suspected CNS IMD may be difficult, as CNS involvement is determined by the inflammatory host response [24,25,26]. For optimal evaluation of infectious lesions, the following sequences must be included: diffusion-weighted imaging (DWI), fluid-attenuated inversion recovery (FLAIR), T2-weighted imaging (T2-W), T2*-weighted imaging (T2*), T1 before and after contrast (T1+C). In addition, the importance of susceptibility-weighted imaging (SWI) was recently reported, as SWI is more sensitive than T2* in outlining areas of hemorrhage and the venous structures [24].

### 5.1. Fungal Sinus Disease

In immunocompromised patients, fungal sinus disease can present as nonspecific mucosal thickening or as a fulminant infection with vascular invasion [27], and acute invasive fungal rhino-sinusitis; which is the most urgent and severe form with a mortality of 50–80% [28]. The infection commonly begins as mucosal inflammation and rapidly spreads to the maxillary, ethmoid, or sphenoid sinus, usually affecting multiple, but unilateral sinuses (Figure 1A,B) [29]. The infection may spread to the adjacent soft tissue and orbits with subsequent destruction of the sinus walls and cribriform plate (Figure 1C) and extension to the orbits and/or anterior cranial fossa. The CNS may be affected through direct extension or, due to the angio-invasive nature of the fungus, through vascular invasion. It might be helpful to perform a CT scan to detect areas of destructed bones (Figure 1C), whereas MRI evaluates better fungal invasion of soft tissue beyond the sinuses to the cavernous sinus and the brain. Rarely, a fungal ball can be detected, which appears on T2-W hypointense (Figure 1A) due to the absence of water and with a hyperintense-inflamed mucosal lining.

### 5.2. CNS Manifestations

**Figure 2:**
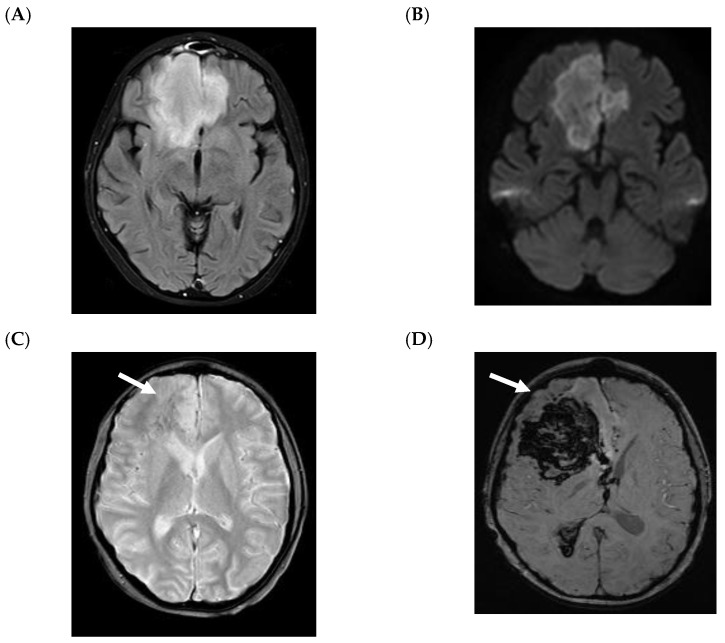
A 12-year-old boy with acute lymphoblastic leukemia with frontal cerebritis in the right and left frontal lobe and probable central nervous system (CNS) invasive mold infection. *Aspergillus fumigatus* and *Rhizopus arrhizus* were isolated outside the CNS. The patient is alive with residual left-sided hemiparesis. A, B and C: Imaging performed during the early stage of frontal cerebritis, mainly on the right side. Fluid-attenuated inversion recovery (FLAIR) image shows hyperintense white matter of frontal lobe (**A**). Diffusion-weighted image (DWI), b = 1000 s/mm^2^, shows diffusion restriction in the area of frontal cerebritis (**B**). T2* image (**C**) shows an ill-defined hypointense area (arrow). No enhancement was seen. After 20 days, magnetic resonance imaging was performed after clinical worsening and suspected vascular involvement (**D**). Susceptibility-weighted image (SWI; D) shows marked decreased signal intensity and blooming with mass effect. Findings were consistent with major secondary hemorrhage (D, arrow) and intraventricular bleeding.

**Figure 3:**
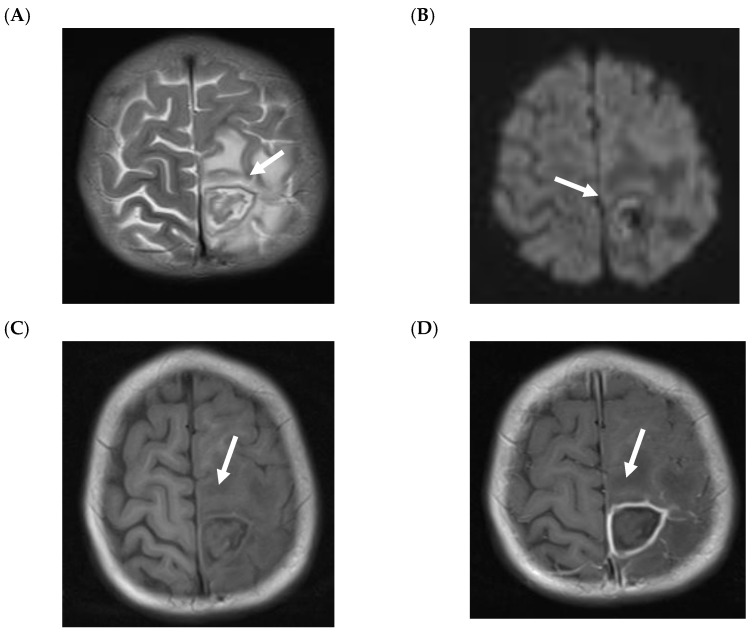
Fungal abscess in a 3-year-old girl with acute lymphoblastic leukemia and proven central nervous system invasive mold infection. The patient is alive with residual right-sided weakness. Axial T2-weighted (**A**) shows a mass in the left central region, which is predominantly of low T2 signal intensity, centrally with hyperintense perilesional edema and a well-defined ring-like hypointense area (arrow). Diffusion-weighted image (DWI), b = 1000 s/mm^2^, shows mainly restriction within the hypointense rim (**B**, arrow). Axial unenhanced T1-weighted shows a slight rim-like high signal intensity probably due to the presence of iron, manganese or methemoglobin (**C**, arrow). T1 after contrast shows a strong ring enhancement (**D**, arrow).

Children with CNS IMD can present with cerebritis and/or abscess formation. The MRI findings in children with cerebritis are nonspecific, but are usually hyperintense in T2-W and FLAIR with variable enhancement (e.g., no, minimal or clear enhancement) (Figure 2) [24]. On T2-W and FLAIR images, it might be difficult to distinguish between cerebritis and perilesional edema secondary to abscess or bleeding (Figure 2A and Figure 3A). Isolated cerebritis in the absence of abscess or CNS-bleeding (with minimal or no enhancement) is very rare in children. Ring enhancement and restricted diffusion are often seen children with fungal abscess (Figure 3) [24]. Ring-like enhancement, which is often observed, is usually associated with perilesional FLAIR- and T2-hyperintensity and might be interpreted as edema with probable infiltration of the adjacent brain parenchyma and/or vasogenic edema (Figure 3A).

Although not specific for fungal pathogens, multifocal ring enhancement and diffusion restriction in immunocompromised children suggest infection. Still, the differentiation between a parenchymal involvement of CNS IMD from other CNS manifestations, such as bacterial infections or tuberculosis, can be difficult.

**Figure 4:**
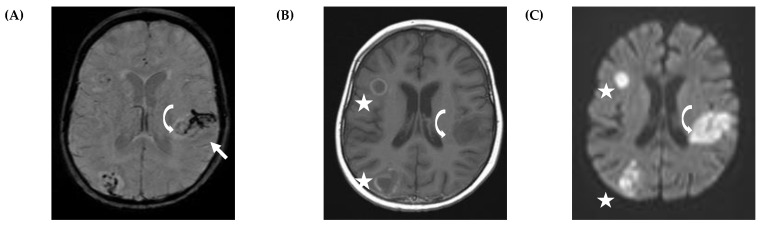
Vascular involvement in a 3-year-old girl with acute lymphoblastic leukemia (ALL) and proven central nervous system invasive mold infection (CNS IMD). The patient is alive but is a wheelchair user. Immunocompromised children have an impaired host immune system to fight against fungi (Figure 4A, curved arrow), which often results in an infiltration of the vascular structures (Figure 4A, straight arrow). Susceptibility-weighted image (SWI) (**A**) shows ring-like fungal infection on the left side (curved arrow) and thrombosis of the deep medullary veins (straight arrow), which are visualized as low signal intensity at SWI. Enhanced T1W (**B**) shows multiple ring-enhancing fungal abscesses on the right (star), but no enhancement on the left side. Diffusion-weighted image (DWI) (**C**) shows restricted diffusion within multiple ring-enhancing fungal abscesses on the right (star). The infarct with diffusion restriction (C) on the left involves the nonenhancing ring-like structure shown in the SWI (A, curved arrow).

The MRI changes of a fungal abscess are similar to a pyogenic abscess as both demonstrate hypointense ring-like changes in T2-W with restricted diffusion (Figure 3A,B) [30]. The pattern of diffusion restriction of the wall without the center correlates with a fungal abscess rather than a pyogenic abscess (Figure 3B. Bacterial and tuberculous abscesses typically demonstrate central restricted diffusion. Nevertheless, it was recently reported that fungal abscesses in children are often associated with diffusion restriction at the core of the lesion (Figure 4), and that classical differential using DWI seems not to be helpful in children [24].

It was reported that abscesses in fungal infections are often multiple and closely located to the grey/white matter junction (Figure 3D and Figure 4B), which results from the hematologic spread of the pathogen [31,32]. In contrast, solitary abscesses are often seen in bacterial infections. In adults, fungal abscesses often affect deep gray matter nuclei, which, however, could not be confirmed in the pediatric population [24,31].

Hemorrhage may complicate the correct diagnosis [24]. On MRI, fungal abscesses are usually hypointense on T1-W image. However, due to the presence of iron, manganese or methemoglobin, areas of high signal intensity on unenhanced T1-W images may be seen (Figure 3C). Due to the hyperintensity in T1-W images, the differential diagnosis with superimposed hemorrhage is difficult.

In contrast to focal reactive meningeal enhancement, diffuse meningitis caused by fungal disease seems to be very rare in children with CNS IMD [24]. Notably, diffuse meningitis associated with invasive fungal infection is seen in immunocompromised adults, particularly in patients with HIV [31].

**Figure 5:**
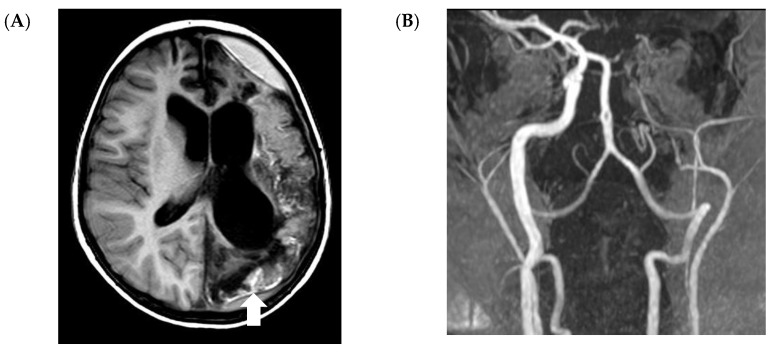
Vascular involvement in a 5-year-old boy with acute lymphoblastic leukemia (ALL) and probable central nervous system invasive mold infection. The patient is alive with residual right-sided hemiparesis. Magnetic resonance imaging (MRI) shows a large hemispheric infarction on the left side and cortical necrosis (arrow) on T1-W images without contrast (**A**). Time-of-flight magnetic resonance angiography (TOF-MRA) (**B**) shows only weak flow of the left internal cerebral artery.

A recent retrospective study in 19 pediatric patients with proven or probable CNS IMD suggested that imaging studies may help to predict outcome [24]. The authors found that in particular vascular involvement (Figure 5), leading to perivascular microbleeding (*n* = 2) and infarction (*n* = 7), is associated with poor outcome, as five of these children died and another two had severe sequelae. Similarly, subependymal enhancement without ventriculitis was observed in two children, and one of them died, whereas the other survived with severe neurological sequelae.

To this end, it is important to mention, that, due to the common dissemination of *Aspergillus* spp., recent pediatric-specific guidelines recommend to perform a cranial MRI in patients with proven or probable pulmonary aspergillosis [23].

## 6. Microbiological Evaluation

**Figure 6:**
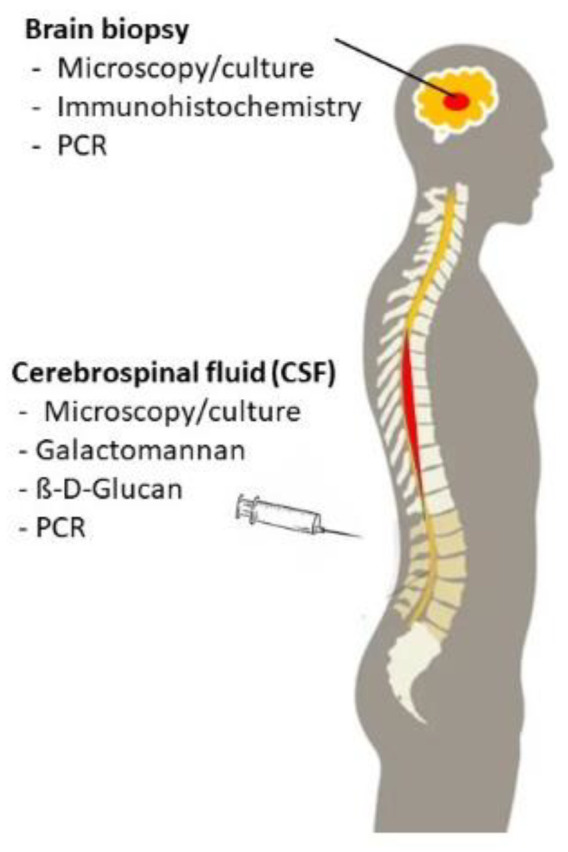
Diagnostic tools for brain biopsy specimens and for cerebrospinal fluid.

In addition to imaging studies, microscopy, culture as well as non-culture-based tests detecting fungal DNA or antigens are important diagnostic tools in immunocompromised patients with suspected CNS IMD. Diagnostic material includes the cerebrospinal fluid (CSF) which is usually easy to obtain. However, CSF remains nondiagnostic in patients with fungal brain abscess who mostly do not suffer from fungal meningitis. In these patients, a biopsy is required to establish the diagnosis of CNS IMD (Figure 6).

### 6.1. Microscopy

Microscopic examination of CSF or a biopsy specimen is rapid to perform and increases the diagnostic yield of culture methods [33]. Microscopic examination may allow a first differentiation of several groups of fungi such as hyaline molds or mucorales based on the morphologic characteristics of fungal structures. However, it is important to note that pretreatment with antifungal compounds can impact on fungal morphology. As Gram- or hematoxylin–eosin staining may be insufficient to detect fungal elements, which will then misinterpreted as artefacts, the use of ‘special fungal’ stains, such as periodic acid–Schiff (PAS) stain and Grocott’s methenamine silver stain, or optical brighteners (fluorescent whitening agents, such as Blankophor or Calcofluor white) are advised [9,33]. In addition to microscopy, immunohistochemistry should be performed, which might have an important clinical impact as it helps to differentiate between *Aspergillus* spp. and *Mucoraceae* [34]. Additionally, fluorescent in situ hybridization (FISH) is an important methodology which allows for the rapid and specific identification of fungal structures [35].

### 6.2. Culturing Techniques

In all immunocompromised patients in whom CNS IMD is suspected, clinical samples such as CSF or biopsy material must be cultured for fungi in order to identify species and determine antifungal susceptibility. Molds are generally more difficult to isolate from these materials than *Candida* spp. [36]. As fungi often do not grow well on conventional media used for the cultivation, specific agar and broth media and specific culture conditions (e.g., culture at 26 °C over a prolonged period of time) may be required. Therefore, the microbiological laboratory should be consulted before material from a patient with suspected CNS IMD is obtained. These samples have to be processed promptly, and, if this is not possible, storage at 4–5 °C is recommended [33]. Importantly, biopsy specimens must be kept moist and should not be placed in histopathology fixatives. If a fungus has been isolated by culture, it should be identified down to the species level which might have an important clinical implication [37]. In addition, susceptibility testing should be performed [36]. Although culture is the gold-standard to diagnose invasive fungal disease, the method is not very sensitive and, therefore, negative culture results do not exclude fungal infection.

### 6.3. Non-Culture-Based Assays

Due to the low sensitivity of microscopy and culture, several non-culture-based assays have been developed to support the diagnosis of IFD. Whereas multiple studies have assessed the value of antigen and PCR assays for blood and bronchoalveolar lavage (BAL) specimens, the value of these assays in CNS samples is less clear.

### 6.4. Galactomannan

Galactomannan (GM) is a polysaccharide cell-wall component that is released by most *Aspergillus* spp. during its hyphal growth, which can be detected by a number of commercially available enzyme immunoassays (EIA) or point-of-care tests [38,39]. The detection of GM in serum, BAL or CSF is included as a mycological criterion in the recently updated and revised definitions of IFD from the European Organisation for Research and Treatment of Cancer/Mycosis Study Group (EORTC/MSG) consensus group [40]. The value of the assay does not seem to be different in children compared to adults [41]. Circulating GM may become positive several days prior to the clinical manifestation of invasive aspergillosis [42], but circulation of GM in serum is transient, and therefore, screening testing should be done at least twice a week [36]. False-positivity has been described by the administration of various antibiotic compounds or the transfusion of antiglobulin or blood, whereas mold-active antifungal prophylaxis significantly decreases the sensitivity of the assay [43,44]. Notably, cross-reactivity has been observed with *Penicillium*, *Histoplasma* or *Fusarium* species [45]. Current guidelines recommend prospective monitoring of serum GM twice weekly for early diagnosis of invasive aspergillosis in children at high risk for IFD who do not receive mold-active prophylaxis (A-II), whereas it is discouraged for patients on mold-active prophylaxis (D-IIt) [23]. GM should be assessed in children with prolonged febrile neutropenia and/or abnormalities in chest CT (A-II) [23]. However, careful interpretation of the test results is necessary due to limitations such as poor positive predictive values which mean that actions based on test results are often incorrect and due to the fact that negative test results do not rule out infections due to non-*Aspergillus* molds. It is also important to note that the GM-assay is not validated in non-neutropenic patients.

Analyses in adults indicate that the performance of galactomannan in BAL is better than that in serum [42]. These data are supported by two pediatric studies [46,47], and the assessment of GM in BAL is recommended as an adjunctive method for diagnosis of invasive pulmonary aspergillosis [23].

Data of the value of GM in the CSF are scarce and consist of retrospective analyses of case reports and small case series only. A study in 17 patients with probable and proven cerebral aspergillosis, of whom three were younger than 18 years of age, reported that GM in the CSF was positive with an optical density (OD) > 2.0 in 15 patients, whereas 26 out of 27 patients without CNS aspergillosis had a negative GM defined by an OD < 0.5 [48]. Imaging studies revealed multiple brain lesions in most of the patients, only one patient had signs of meningitis. Another study analyzed 15 children with a median age of 16.3 years (range, 3.1–18) who suffered from proven or probable CNS IMD [49]. In nine out of these 15 patients, GM was measured in the CSF, with five having positive and four having negative results (OD ≥ 0.5). CSF culture remained negative in all the seven patients in whom culture was performed in parallel. GM in the CSF was the only positive microbiological marker in three of the 15 patients. In all 32 children without CNS IMD who served as control, GM in the CSF remained negative. Although GM is not validated in the CSF and available data are very limited, the assessment of GM in CSF is recommended as adjunctive diagnostic method in immunocompromised patients with suspected CNS IMD [23]. The threshold of the OD in the CSF should be 0.5.

### 6.5. Beta-D-Glucan

Beta-D-Glucan (BDG) is a polysaccharide fungal cell wall component of various fungi such as species of *Aspergillus*, *Candida*, *Fusarium* or *Pneumocystis jirovecii*, but is absent in patients with cryptococcosis and mucormycosis [50]. Importantly, BDG can be detected in patients with bacterial infection due to *Streptococcus pneumoniae* or *Pseudomonas aeruginosa*, but also in healthy individuals, where levels are higher in children than in adults [51,52]. There are several commercially available assays which use different thresholds. A number of factors may result in false positive results, such as the use of cotton surgical swabs, blood transfusions, albumin and immunoglobulin infusions, hemodialysis or mucositis [53]. Although serum BDG is included in the recently updated definitions of IFD given by the EORTC/MSG consensus group, it is important to note that the marker is not specific for any IFD, and should not be used to rule in patients for clinical trials [40]. A recent Cochrane analysis included 49 studies with a total of 6244 participants in which the clinical utility of serum BDG was tested [54]. Unfortunately, due to the considerable heterogeneity between studies, a formal meta-analysis and an estimation of the value of the test was not possible. Similarly, a pediatric specific meta-analysis demonstrated highly variable sensitivity, specificity, positive and negative predictive values of BDG [41]. Pediatric specific guidelines recently gave a recommendation against the use of serum BDG in clinical decision making (D-II) [23].

Very limited data are reported on the value of BDG assessed in the CSF. One pediatric study reported on serial BDG levels in CSF samples obtained from children aged 1 month to 18 years with a diagnosis of probable or proven *Candida* (*n* = 7) or *Aspergillus* (*n* = 2) CNS infection [55]. In all children, BDG was detected in the CSF, which corroborates an analysis of nine adult patients (median age 47 years, range, 18–97) with proven or probable CNS IFD [56]. As data are too limited, pediatric guidelines mention the assessment of BDG in the CSF without grading [23].

### 6.6. Molecular Test Methods

Although molecular assays for the detection of fungal DNA have been evaluated for more than two decades, the acceptance of this methodology took a long time as tests often differ in the gene sequence detected, the origin of samples (e.g., whole blood, serum), the method of DNA extraction or the primers used (*Aspergillus*-specific PCR versus “pan-fungal”-PCR) [57]. However, methods have now been standardized by the effort of the European *Aspergillus* PCR initiative (EACPRI), and quality controls for molecular diagnostics have been implemented [58]. A systematic review of the literature demonstrated that sensitivities and specificities of GM, BDG and *Aspergillus* PCR were comparable [59]. Based on these facts, the updated definitions of IFD by the EORTC/MSG consensus group included *Aspergillus* PCR performed in blood samples and BAL fluid as microbiological criterion supporting the diagnosis of invasive *Aspergillus* infection [40]. However, due to limited and heterogenous data in the pediatric setting, no specific recommendations have been made for molecular testing methods in children [23]. Interestingly, in contrast to GM testing, mold-active prophylaxis decreases the specificity, but not the sensitivity of *Aspergillus* PCR, which might be due to the fact that mold-active prophylaxis limits the clinical progression of invasive aspergillosis resulting in false-negative GM test results [60]. It has been suggested that the combination of biomarkers for testing blood or BAL samples increases the clinical utility, but this has to be confirmed in large multicenter studies [61,62,63]. Promising results in predicting invasive aspergillosis have been reported for combining genetic risk markers, established clinical risk markers and diagnostic biomarkers [64].

In addition to blood and BAL fluid, molecular methods added to histopathology and culture of tissue biopsy specimens may improve both the detection and specification of fungal pathogens [65,66]. The EORTC/MSG consensus group strongly recommends that specialized laboratories, which are rigorously quality controlled, should be contacted for these tests.

There are extremely limited data on the performance of PCR assays in detecting CNS IFD in the pediatric population. In one retrospective study in mostly adults, *Aspergillus*-specific nested PCR detected CNS aspergillosis in the CSF of all eight patients with proven and probable CNS aspergillosis (median age, 38 years, range 4–84) [67]. As two out of 25 PCR tests in individuals without CNS aspergillosis were positive, sensitivity and specificity values were 1.0 (95% CI 0.68–1) and 0.93 (95% CI 0.77–0.98), respectively. A pediatric specific study included 15 patients with proven and probable CNS IMD, and in eight of these patients, PCR testing was performed (biopsy and CSF specimen in five and three patients, respectively) [49]. In six patients, fungal nucleic acids were detected (*A. fumigatus* in five and *Fusarium* spp. in one patient, respectively), whereas PCR remained negative in two children (biopsy and CSF in one patient each). Microscopy was positive in all four patients in whom cytology was performed, whereas culture remained negative in both patients in whom results of culture were available. In the two patients with negative PCR results, microscopy revealed hyphae. Although the clinical value of PCR testing in CSF specimens is not well established in the pediatric setting, most experts agree that molecular testing should be performed as adjunctive diagnostic tool, as diagnosing CNS IMD is extremely difficult.

### 6.7. MALDI-TOF Mass Spectrometry

In many laboratories, microbial species are identified by the purely biophysical and bioinformatics-driven matrix-assisted laser desorption/ionization time-of-flight mass spectrometry (MALDI-TOF MS). The advantage of this method is the higher precision compared to biochemical and microscopic methods, as well as the quick turn-around time. However, the classification relies on extensive well curated databases [68]. In brief, chemically ionized cellular contents serve as the analytes, which are desorbed from the target plate by laser fire and accelerated in an electric field along an evacuated flight tube. Time-resolved impact on a detector yields a characteristic biomarker mass spectrum, which can be used to search a database of reference entries [68]. In order to precisely identify a specific species, the observed masses need to be highly reproducible within isolates of the same species, but also need to be sufficiently different between highly related species [68]. The method is well established for the identification of yeasts, and has considerably been improved over the last years for molds which are phylogenetically closer related.

## 7. Therapy and Outcome

Treatment Recommendations

As children differ from adult patients in a number of conditions, such as in host biology, cancer types, treatment and comorbidity, as well as in the approval and dosing of antifungal agents (Table 1), there is growing interest in establishing pediatric specific guidelines [69].

Recent pediatric guidelines recommend voriconazole, and, with a lower grade, liposomal amphotericin B for targeted treatment of invasive aspergillosis (A-It and B-It, respectively) [23]. In CNS aspergillosis, there is a clear preference for voriconazole. Of note, voriconazole is not approved for children younger than 2 years, and therapeutic drug monitoring is strongly recommended. Liposomal amphotericin B is approved for children of all ages. Although it is well known that CSF concentrations after the intravenous application of a colloidal dispersion and of several liposomal preparations of amphotericin B are low, there is a large body of clinical experience of the successful use of different amphotericin B preparations for CNS infections in humans [70]. Notably, flucytosine has not been included in the guidelines for the treatment of CNS aspergillosis, although in vitro data suggested a clinical benefit [23,71].

For the treatment of mucormycosis, the strongest recommendation is given for high-dose liposomal amphotericin B (A-II; recommended dosage 5–10 mg/kg), whereas amphotericin B lipid complex and combination therapies which include liposomal amphotericin B have a lower recommendation (B-II and C-III, respectively) [23]. The broad-spectrum triazoles posaconazole and isavuconazole are not licensed in the EU for pediatric patients, and are listed without grading in the pediatric specific guidelines for the treatment of mucormycosis [23]. The efficacy of posaconazole solution was established in adults [72,73], but in the pediatric setting, the target level between 500 and 2500 ng/mL was not achieved by most of the subjects [74]. Recently, a novel powder for oral suspension (PFS) was developed which offers a better bioavailability, and for both PFS and intravenous solution pediatric dosages have been established [75]. However, the penetration of posaconazole in the CNS is poor [70]. Isavuconazole is a novel triazole with broad-spectrum activity which has shown good blood–brain-barrier penetration. A recent retrospective analysis of 36 adult patients with CNS invasive fungal disease (half of them due to *Aspergillus* spp. and mucorales) reported on an excellent overall survival of 80.6% on day 42 [76]. Although a retrospective study including 29 immunocompromised children receiving isavuconazole off-label suggested the usefulness of the compound in the pediatric setting, data of isavuconazole in children are very limited [77]. As there is a lack of pediatric data regarding the treatment of rare molds, such as *Fusarium* spp. and *Scedosporium* spp., pediatric guidelines are based on adult experience. For fusariosis, treatment with voriconazole or liposomal amphotericin B is recommended. For treatment of *Scedosporium* spp., which have variable susceptibility to itraconazole, voriconazole, posaconazole, and micafungin, no solid recommendation can be made [78]. Notably, the ability of the echinocandins inlcuding micafungin to penetrate the blood–CSF and the blood–brain barrier is poor as a consequence of their high molecular mass [70].

Although reported for some patients [79,80], experts do not recommend intrathecal or ventricular administration of antifungal agents due to the lack of proven efficacy and the risk of local toxic effects [13].

In addition to the prompt initiation of antifungal therapy, both pediatric and adult guidelines recommend the control of predisposing conditions (e.g., colony-stimulating factors in neutropenia, reduction of immunosuppressive therapy, in particular steroids) [23]. Whether hyperbaric oxygen, as used in some children with CMS mucormycoses, has any clinical benefit is unclear to date [79,80].

Surgery plays an important role in therapy, in particular in patients with rhino-sinusitis form of mucormycosis, but surgical interventions should be discussed on a case by case basis using a multidisciplinary approach [81]. In this regard, a case series of four children with leukemia suffering from CNS aspergillosis (single cerebral abscess and multiple fungal abscesses in two patients each) reported that after frameless stereotactic resection, three patients were alive without evidence of CNS aspergillosis after a follow-up of 2–4 years, whereas one patient died of leukemia [82]. However, as patients are usually treated with a multimodal approach including surgery and the administration of antifungal compounds, the exact role of surgery cannot be defined [79].

The overall mortality of CNS IMD has decreased over time, which might be due to factors such as improved diagnostic tools and the availability of potent antifungal compounds. A systematic review including patients up to 2005 found a mortality of 82.8% of CNS aspergillosis reported before 1990, while that of patients reported after 1990 was 39.5% [83]. In a recent analysis on immunocompromised children suffering from mucormycosis, five out of eight patients (62.5%) with rhino-cerebral involvement died, but it has to be noted that the outcome of invasive aspergillosis is generally better than that of mucormycosis [10,11]. Another analysis of 29 immunocompromised children diagnosed with CNS IMD between 2007 and 2016 reported that the probability of 2-year survival was 48.9% [22]. Unfortunately, only four of 14 children undergoing HSCT were alive after 2 years, whereas this was the case in 11 out of 15 patients receiving chemotherapy. However, almost half of the survivors suffered from severe neurological sequelae such as hemi-/quadriplegia, visual impairment or aphasia.

## 8. Conclusions and Perspectives

Invasive mold disease of the central nervous system (CNS IMD) is associated with significant morbidity and high mortality. The clinical presentation of CNS IMD is uncharacteristic, and, in the early stage, the infection might be asymptomatic. Imaging, in particular magnetic resonance imaging, is an important diagnostic tool, but findings are uncharacteristic and often difficult to interpret. New strategies such as labeled antibodies may help to increase both sensitivity and specificity of diagnostic imaging in the future [84]. In addition, nonculture based microbiologic assays such as antigen detection and molecular tests need to be validated in CNS specimens, and their clinical value has to be established in the pediatric setting. Although new and potent antifungal compounds helped to improve outcome of CNS IMD, not all agents are approved for children and a pediatric dosage has not been established. Therefore, studies have to rapidly evaluate dosage, safety and efficacy of antifungal compounds in the pediatric setting. In addition, it has to be assessed whether immunotherapeutic approaches will help to decrease long-term sequelae and mortality of CNS IMD in children.

## Figures and Tables

**Table 1:** Antifungal agents for potential use in invasive mold disease of the central nervous system (CNS).

Compound (Formulation)	Pediatric Approval	Pediatric Dosage	Comments
Liposomal amphotericin B (iv)	Children of all ages	3 mg/kg per day in one dose Mucormycosis: 5–10 mg/kg per day in one dose	Less nephrotoxic than amphotericin B deoxycholate
Amphotericin B lipid complex (iv)	Children ≥ 1 month	5 mg/kg per day in one dose	Infusion-related toxicity similar, but less nephrotoxic than amphotericin B deoxycholate
Voriconazole (iv and oral)	Children ≥ 2 years	Children aged 2–<12 years or 12–14 years and weighing <50 kg: 8 mg/kg (day 1, 9 mg/kg) twice daily intravenously or 9 mg/kg twice daily orally; children aged ≥15 years or 12–14 years and weighing ≥50 kg: 4 mg/kg (day 1, 6 mg/kg) twice daily intravenously or 200 mg twice daily orally	TDM recommended First choice in CNS aspergillosis Not active against mucormycetes
Posaconazole (iv and oral)	EU: no US: ≥13 years (prophylaxis)	Patients ≥13 years: Delayed release tablets, 300 mg/d in one dose (day 1: 300 mg twice); patients from 1 month to 12 years: oral suspension, starting dose 6 mg/kg three times daily + TDM	Problems with absorption with the oral suspension Most likely limited activity in CNS
Isavuconazole (iv and oral)	No	10 mg/kg per day in one dose, maximal dose of 372 mg isavuconazonium sulfate (d 1 and 2: three times daily)	Suggested dose corresponds to that investigated in a pediatric phase II trial (part of the Pediatric Investigation Plan)
Caspofungin (iv)	Children of all ages	50 mg/m^2^ per day (day 1, 70 mg/m^2^) in one dose (maximum dose 70 mg per day)	Limited penetration to the CNS
Micafungin (iv)	Children of all ages	2–4 mg/kg per day (in children weighing ≥50 kg, 100–200 mg) in one dose	Limited penetration to the CNS

iv, intravenous; TDM, therapeutic drug monitoring; CNS, central nervous system.
